# One independent or many independent? The relationship among self-construal, number of brand endorsers, and brand attitudes

**DOI:** 10.3389/fpsyg.2024.1328281

**Published:** 2024-02-02

**Authors:** Shichang Liang, Kunhan Cai, Yiwei Zhang, Xueying Yuan, Siyu Pan, Lili Teng

**Affiliations:** ^1^School of Business, Guangxi University, Nanning, Guangxi, China; ^2^China-Asean Institute of Financial Cooperation, Guangxi University, Nanning, Guangxi, China; ^3^Business School, Central South University, Changsha, Hunan, China

**Keywords:** brand endorser, number of endorsers, self-construal, brand attitude, self-consistency

## Abstract

**Introduction:**

It was common for brands to use different numbers of endorsers in marketing practice. Nevertheless, research on brand endorsers’ quantity has not yielded a uniform consensus. The previous research about brand endorsers mainly focuses on the appeal of endorsement, brand category, and endorser characteristics, paying less attention to the impact of cultural factors, particularly self-construal. This study delves into selecting brand endorsers across diverse cultural regions for the same brand.

**Methods:**

Drawing on the principles of self-consistency theory and self-construal theory, our research, conducted through three distinct experiments, reveals that consumers tend to hold more favorable opinions about brands endorsed by a single individual. Furthermore, self-consistency emerges as a crucial mediating factor in this phenomenon. Additionally, self-construal is an essential factor among consumers from various cultural backgrounds.

**Results:**

Consumers with an independent self-construal exhibit more favorable brand perceptions when it comes to single-endorser brands compared to their counterparts with an interdependent self-construal. Conversely, individuals with an interdependent self-construal demonstrate a more positive disposition towards brands with multiple endorsers than those with an independent self-construal.

**Discussion:**

This research not only enriches and extends our theoretical understanding of the impact of the number of brand endorsers on consumer brand attitudes but also provides valuable practical insights for optimizing the selection of brand endorsers for companies.

## 1 Introduction

Brand endorsers usually represent the brand’s image and connotation and bring additional commercial value to the brand (Elberse and Verleun, 2012; Tian et al., 2022; Yang et al., 2022). Further, with the rise of the fan economy, brands are increasingly emphasizing their choice of endorsers’ strategies (Lipsman et al., 2012; Teng et al., 2020), such as the number of endorsers. Nowadays, it has become common for brands to use different numbers of endorsers in different regions. Some companies find it more effective to use multiple brand endorsers, such as CHANEL’s choice of 9 brand endorsers from different countries and regions in 2023. Some companies believe that endorsements are about quality rather than quantity. For example, Dong Mingzhu is not only the chairman of Gree Electric, but she also became the sole endorser for Gree Electric herself in 2012 and remains the sole endorser today. Some companies choose different numbers of endorsers to face different regional markets. In 2020, OPPO chose 10 endorsers to face the Chinese market. However, when facing the Middle East and Africa market, it chose only one endorser, Liverpool Salah.

On the other hand, with the metaverse and the digital economy becoming a global trend, virtual endorsers have become a hot topic for companies and consumers. Also, adding novelty to an advert with a virtual digital human endorsement is more accessible than with a natural person endorser (Franke et al., 2023). Similarly, companies will consider selecting one or more virtual endorsers for their endorsement campaigns. Virtual endorsers are often brand mascots or virtual digital human. For example, after digitizing its mascot, MIXUE announced that the Snow King would be the sole global endorser of the brand; Bilibili has chosen two virtual endorsers, “22” and “33,” to be its global endorsers. Moreover, ASUS launched “Princess Tianxuan” as the sole virtual endorser in March 2020 and added “SE7EN” as the second virtual endorser in October of the same year.

So, does choosing different numbers of endorsers based on individual consumer characteristics in different cultural markets, as OPPO did, produce different marketing effects? Does this effect also arise when a virtual digital human endorsement is made? These are the questions that the research in this paper will explore.

Numerous research efforts have explored the phenomenon of brand endorser quantities. Prior scholars have investigated individual endorsements, focusing on the appeal associated with a single celebrity endorser (Yang et al., 2022), the number of brands endorsed by one celebrity endorser (Zhu et al., 2019), and the types of individual endorsers (McCormick, 2016; Schouten et al., 2020). On the other hand, studies on multiple endorsers have been conducted by previous scholars regarding the type of combined endorsement (Amason, 1996) and celebrity familiarity of multiple endorsers (Thomas and Fowler, 2015; Von Felbert and Breuer, 2022). In addition, some scholars have also studied both single endorsers and multiple endorsers, focusing on consumer engagement (Handriana, 2017) and the number of brands (Tripp et al., 1994). Nevertheless, research on brand endorsers’ quantity has not yielded a uniform consensus. It has predominantly centered on variables such as the appeal of endorsement, brand category, and endorser characteristics, paying less attention to the impact of varying cultural factors, particularly self-construal. This study delves into the role of cultural variables, specifically self-construal, in shaping the number of brand endorsers and consumers’ perceptions of brands.

Self-construal pertains to how an individual perceives their connection with others, a perception that is influenced by their cultural heritage. It can be categorized into two distinct types: independent self-construal, emphasizing individualism, and interdependent self-construal, highlighting collectivism (Markus and Kitayama, 1991). Individuals characterized by an independent self-construal prioritize product uniqueness in their purchasing decisions, whereas those with an interdependent self-construal emphasize product conformity (Lee and Shavitt, 2004). When a brand receives an endorsement from a single individual, consumers tend to associate it with the characteristics of that person. In contrast, a brand endorsed by multiple individuals is often seen as embodying the traits of a collective (Ran et al., 2020). This suggests that independent self-construal consumers may be more drawn to single-endorsed brands, while interdependent self-construal consumers may prefer multi-endorsed brands. Therefore, this article proposes that independent self-construal consumers exhibit more positive brand attitudes toward single endorser brands than interdependent self-construal consumers. In comparison, consumers with an interdependent self-construal showed more positive attitudes toward brands with multiple endorsers than consumers with an independent self-construal.

This paper utilizes self-consistency theory and self-construal theory to explore the impact of self-construal on the relationship between the number of brand endorsers and brand attitudes. Three experiments will be conducted to gather data and test these hypotheses.

## 2 Conceptual framework

### 2.1 Brand endorser

Brand endorsers can build a positive image for a brand and symbolize the brand’s unique personality (McCracken, 1989). Consumers tend to perceive trustworthy endorsers as providing more reliable information and having more favorable attitudes and purchase intentions toward the brands they endorse (McGinnies and Ward, 1980). Therefore, endorsers are vital in shaping consumers’ brand perceptions.

Previous scholars have mainly studied consumer psychology from the endorser’s characteristics, the connection between endorser and brand, celebrity endorsements and influencer endorsement. First, from the study of the personal traits of brand endorsers, the gender traits of endorsers (masculine performance vs. feminine performance) (Worth et al., 1992) and the personality traits of endorsers (self-improvement vs. self-deprecation) can affect consumers’ impressions of brands and products (Kozinets et al., 2010) and the temperature of the endorser’s expression (Xie et al., 2019) can affect consumers’ impressions of brands and products. Consumers from different cultures differ in their preferences for the personal characteristics of endorsers. In China (vs. the West), endorsers with smart traits (vs. sexy) are better at raising positive consumer attitudes toward luxury products and brand advertising (Yin et al., 2019).

Secondly, the relationship between the endorser and the brand also impacts consumer attitudes. The better the match between the endorser and the endorsed product, the more influential the endorsement will be (Schouten et al., 2020). The word-of-mouth communication of the endorser in the brand community will influence the relationship and trust between consumers and the brand (Banerjee and Sreejesh, 2022). On social media, fans follow and unilaterally interact with endorsers they do not personally know, which is called “parasocial interaction” (Giles, 2002; Thorson and Rodgers, 2006). Even if such interactions are “one-sided psychological engagement” and “illusive mutual awareness,” (Hartmann and Goldhoorn, 2011) they can enhance consumers’ positive attitudes and purchase intentions (Gong, 2021).

Finally, celebrity and influencer endorsements, as commonly used endorsement strategies, also impact consumer attitudes. Positive celebrity messages positively impact purchase intentions, brand attitudes, and consumer attitudes toward advertising (Amos et al., 2008). Specifically, celebrities will transfer their social and cultural significance to the brand or product they endorse, thus promoting consumer behavior based on celebrity identification, which is the celebrity effect (Erdogan and Baker, 2000). Not merely that, the “celebrity effect” also increases the market demand for the product (Newman et al., 2011). Some scholars have also studied distinctive poses in celebrity endorsement commercials, suggesting that when celebrities use distinctive poses in endorsements, it is easier to increase consumer attitudes toward the brand (Liu and Liu, 2019). Celebrity marketing is always an influential area of research in marketing, with different research priorities at different times. However, with the increased influence of social media, the focus of companies seeking endorsements is gradually shifting from celebrities to social media influencers (Ozdemir et al., 2023). As for research on social media influencer endorsements, recent studies have focused on virtual influencer endorsements (Wang and Liu, 2023). Technological advances brought about by the Fourth Industrial Revolution and the popularity of social media influencers have led to the popularity of “virtual influencers” on social networks (Qu and Baek, 2023). Virtual influencers are digital characters created by computer graphics software and given a personality by their production team. With the ability to publish and impact media platforms, virtual influencers are prompting more and more corporations to start applying virtual influencers as an alternative to human influencers in the digital age (Ozdemir et al., 2023).

### 2.2 Number of brand endorsers

Studies on the impact of the number of brand endorsers on consumer attitudes have yielded diverse findings. Research on single celebrity endorsements has shown that the physical attractiveness of a celebrity influences consumer attitudes and purchase intentions (Kahle and Homer, 1985). However, increasing celebrity endorser brands can negatively affect consumers’ brand attitudes (Tripp et al., 1994). Furthermore, a lower suitability of the endorser’s temperament type to the brand’s connotations can also lead to a decline in brand appeal (Zhu et al., 2019). On the other hand, research on multiple endorsements has also focused on the characteristics of the endorsers. Studies on multiple endorsements show that the effectiveness of the number of endorsers depends on the perceived fit between the features of multiple endorsers (Wang et al., 2015). Multiple endorsements are more beneficial to consumers’ brand attitudes than single endorsements, while for high-engagement products, single endorsements are more beneficial than multiple endorsements (Rice et al., 2012; Handriana, 2017).

In conclusion, previous research has focused primarily on the impact of the attributes of a single endorser or multiple endorsers on the effectiveness of brand endorsement, with less attention paid to the impact of consumers’ cultural predispositions on preferences for the number of brand endorsers. In addition, prior studies have not yielded uniform results on whether single or multiple endorsers lead to more favorable brand attitudes. For example, previous scholars have discussed the impact of the number of brand endorsers (single vs. multiple) on brand attitudes in terms of the level of consumer involvement and have come up with different results. Some scholars concluded that multiple endorsers (vs. single endorsers) resulted in more positive brand attitudes when consumer involvement was low (i.e., unfamiliar with the product) (Saleem, 2007; Rice et al., 2012; Handriana, 2017). Other scholars have obtained the opposite result, suggesting that single endorsements (vs. multiple endorsements) lead to more positive brand attitudes at low levels of involvement (Thomas and Fowler, 2015). Therefore, this study aim to addresses this gap and explores the effects and underlying mechanisms of the number of endorsers on consumer brand attitudes.

Celebrity endorsement is one of the most common endorsement strategies used by brands. Celebrities can convey the meaning of the endorsed brands to consumers through endorsement activities (Miller and Allen, 2012). Consumers self-construal the brand and product meanings conveyed by celebrity endorsements into their attributes, thereby creating a new self-image (McCracken, 1989). Brand managers position their brands through celebrity endorsement and create a personified image for the brand with the relationship of celebrity-brand dependency, thus allowing consumers to associate the celebrity’s personality with the brand’s personality (Arora et al., 2021). The celebrity’s image is transferred to the brand image and significantly impacts consumers’ perception of the brand. A celebrity endorsement transfers the qualities of a single individual to the brand, while multiple celebrity endorsements transfer the qualities of a group to the brand. Attribution theory suggests that trait inferences may lead consumers to evaluate multiple endorsers less than single endorsers (Kelley, 1973). In addition, Sears (1983) argued that according to person-positivity bias, people evaluate people who are like themselves more positively; thus, they do not give positive evaluations to groups who are less like them. Previously, scholars have found that individuals are nervous and anxious about facing groups in real life, while individuals feel relaxed and open when faced with individuals (Barasch and Berger, 2014).

Besides, multiple endorsement ads bring more visual complexity. Visual complexity can pose challenges for consumers in their decision-making process (Wedel and Pieters, 2015). It can compromise the quality of their decisions when faced with excessive and complex information (Vogrincic-Haselbacher et al., 2021). When overwhelmed with information, consumers tend to adopt simple decision-making strategies (Grether and Wilde, 1983) and prefer single endorser brands which are easy to process information. The primacy effect, proposed by Asch in 1946, emphasizes the effect of “first impressions.” Specifically, in the impression formation process, the order in which information appears has an important influence on impression formation. Compared with the information that appears later, the information that appears first will make a deeper impression. Therefore, since most of the endorsements of a brand are continuously added each year, influenced by the primacy effect, people tend to be more impressed by the first endorser and less impressed by the multiple endorsers that follow. From that point of view, a single endorsement helps to strengthen consumers’ impression of the brand and create a good brand attitude. In contrast, the significance of multiple endorsements for a brand is then invalidated. The following hypothesis is proposed:

*H1*: Single endorsements lead to more positive brand attitudes than multiple endorsements.

### 2.3 Number of brand endorsers and self-construal

Markus and Kitayama (1991) state that self-construal is defined as an individual’s perception of his or her relationship with others under different cultural influences. According to individualistic and collectivistic cultures, it can be divided into two perspectives: independent self-construal and interdependent self-construal. Individualistic cultures emphasize the significance of each person as an independent individual, and individuals will view the self as a bounded, unique, and primarily stabilized motivational and cognitive system. For example, Westerners who emphasize an individualistic culture tend to define the self-based on their achievements and eminence. In contrast, collectivistic cultures emphasize the role of interpersonal relationships and people maintaining interconnectedness and dependence on each other. Thus, East Asians who emphasize collectivistic cultures tend to define the self-more by defining the self in relation to others.

Many studies prove that independent and interdependent self-construal are not completely separated and can co-exist in one body, but one self-construal will be dominant in a particular situation (Brewer and Gardner, 1996). Research on self-construal has mainly focused on its social dimensions, such as its impact on creativity (Jin et al., 2016) and resource allocation (Murphy-Berman et al., 1984). These studies highlight the importance of self-construal as it provides various perspectives for research.

Self-construal holds an important place in the field of consumer behavior. Studies have established the connection between self-construal and consumer behavior (Aaker and Lee, 2001; Shavitt et al., 2009), with independent and interdependent self-construal having a significant impact (Sugitani, 2018). Hence, self-construal is often considered as a key factor when exploring the factors that influence consumer attitudes and behaviors, including brand perception, choice, and evaluation (White et al., 2012), cross-cultural advertising’s effect on brand emotions (Schouten et al., 2020), the impact of self and public evaluations on brand purchase (Sugitani, 2018), and others. However, fewer studies have combined self-construal with the number of brand endorsers. As a cultural variable, self-construal influences consumers’ consumption preferences in different cultural regions. Therefore, this paper explores the effect of the number of brand endorsers on consumers’ brand attitudes from the perspective of self-construal theory.

Previous scholars have argued that different self-construal led consumers to have different consumption goals (Aaker and Lee, 2001). Consumers tend to engage in behavior that matches their self-construal (Belk, 1988), such as purchasing products that match their identity (Ward and Broniarczyk, 2011). Thus, self-construal type affects consumer preferences. For consumers, a brand image that matches their self-construal type is more credible (Agrawal and Maheswaran, 2005). In addition, the number of endorsers also affects consumers’ perceptions, as a brand with a single endorser will have a “single person” quality. In contrast, a brand with multiple endorsers will have a “multiple person” quality (Ran et al., 2020). As people are unique, “single person” traits tend to be individual and unique (Islam and Hussain, 2022); multi-person endorsements match multiple connotations of the brand, so “multi-person” traits tend to be diverse and interrelated. When consumers are independent self-construal individuals, there is a greater emphasis on independence and stability (Markus and Kitayama, 1991). Independent self-construal requires a more unique self-concept than interdependent self-construal (Kampmeier and Simon, 2001). Therefore, we infer that consumer with independent self-construal are more inclined to single-person brand endorsement. In contrast, consumers with interdependent self-construal are more focused on connections with others (Markus and Kitayama, 1991). They prefer product convergence and herding in their consumption behavior (Atakan et al., 2014). Moreover, interdependent consumers also prefer view things from a relational perspective (Kühnen et al., 2001). Therefore, we infer those interdependent self-construal consumers are more inclined to multiple endorsement brands. The following hypothesis is proposed:

*H2*: The self-construal moderates the effect of the number of brand endorsers on consumers’ brand attitudes. Specifically, independent self-construal consumers prefer single endorsements, while dependent self-construal consumers prefer multi endorsements.

Self-concept, an individual’s overall perception of themselves, plays a significant role in this relationship. According to Sirgy (1982), self-consistency is an important aspect of self-concept and influences consumers’ purchase intentions. For example, consumers with higher degrees of self-consistency tend to have stronger purchase intentions (Dolich, 1969). Self-consistency also affects consumers’ brand image. Higher degrees of congruence between consumers’ self-concept and the brand image leads to more favorable product attitudes and purchase intentions (Maille and Fleck, 2011). Furthermore, Zinkhan and Hong (1991) found that consumers prefer brands that align with their true selves in private and social self-concepts in public settings. Thus, self-consistency has a strong correlation with consumers’ brand image.

Brand image affects consumer attitudes toward the brand. Brand image will affect consumers’ willingness to buy (Islam and Hussain, 2022), and brands hope to establish a stable relationship with consumers (Akram et al., 2011). Brands can act as symbols of self-actualization and connect strongly with consumers’ self-concept when they align with their goals (Escalas and Bettman, 2005). The similarity between a consumer’s perceived self-image and the image of brand endorsers can impact their attitudes (Albert et al., 2017). The relationship between a consumer’s self-concept and brand attitudes is established through self-consistency, the agreement between their self-concept and product attributes and image (Sirgy, 1982). Therefore, when consumers associate brands with their self-concept (Dolich, 1969) and are more likely to buy products with a brand image that aligns with their self-concept (Sirgy, 1982). Additionally, there is consistency in the behavior and characteristics of the brand’s target consumer groups (Hamilton and Sherman, 1996). Consumers construct their self-identity and present themselves to others through brand choices based on the consistency between brand-user and self-image associations (Escalas and Bettman, 2003). Both the brand image and the consumer’s self-concept influence their perception of the brand (Aaker and Lee, 2001), and the effectiveness of a brand endorser depends, in part, on the congruence with the brand (Joseph, 1982). As the match between these two factors increases, self-congruence produces a more vital brand state (Aguirre-Rodriguez et al., 2012). Hence, self-consistency positively affects consumers’ brand attitudes (Albert et al., 2017).

It is evident that (1) the number of brand endorsers creates a different brand image. A brand with a single endorser has a personal touch. In contrast, a brand with multiple endorsers has a collective feel. (2) Consumers are drawn to brands that align with their self-concept, and self-conformity enhances their brand attitudes (Lafferty and Goldsmith, 1999). It can even improve their self-esteem in challenging situations (Escalas and Bettman, 2005). Consequently, consumers prefer brands consistent with their traits, i.e., brands that are self-consistency with consumers. As a result, self-consistency mediates the impact of the number of brand endorsers and self-construal on consumers’ brand attitudes. Independent self-construal consumers have a positive attitude toward brands with a single endorser due to self-consistency, while interdependent self-construal consumers prefer brands with multiple endorsers. Based on this, the following hypothesis is proposed:

*H3*: Self-consistency mediates the relationship between the number of brand endorsers and self-construal on consumers’ brand attitudes.

**Figure 1:**
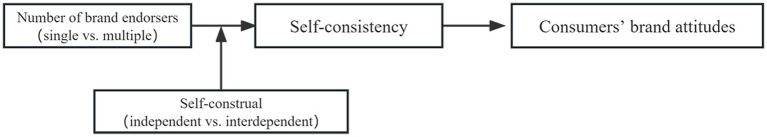
Research model.

The research model is illustrated in Figure 1.

## 3 Study 1

The aim of this study was to test the effect of the number of brand endorsers on consumers’ brand attitudes (H1) and the interactive effect between the number of brand endorsers and self-construal on consumers’ brand attitudes (H2). The study employed a 3 (number of brand endorsers: one person A, one person B, four person) × 2 (self-construal: independent vs. interdependent) between-group design, with one person A being a female endorser, one person B a male endorser, and four endorsers consisting of two female and two male endorsers. Premium cars were chosen as the stimulus and “Benefactor” as the virtual brand to remove the impact of the real brand on consumers. A total of 257 participants were recruited from undergraduate students at a university in southern China, 127 (49.4%) of whom were female, with an average age of 20.71 (SD = 3.177).

### 3.1 Design and procedure

#### 3.1.1 Manipulation of self-construal

This experiment employed a guiding grammar to induce subjects’ self-construal. Based on Trafimow et al.’s (1991) study, the phrase “Please think about what is expected of you” was used for the independent self-construal group, while the phrase “Please think about what is expected of you by your family or friends” was used for the interdependent self-construal group. Participants were given 3 min to reflect and jot down their answers.

#### 3.1.2 Variable measures

Brand attitudes were assessed using Chattopadhyay and Basu’s (1990) brand attitude scale, which consisted of questions such as “I like the brand,” “I approve of the brand,” “I think the brand is good,” and “I am satisfied with the brand.” Responses were recorded on a 7-point Likert scale, with 1 indicating “totally disagree” and 7 indicating “totally agree.”

#### 3.1.3 Experiment procedure

Subjects were randomly divided into six groups: independent endorsement by one male endorser, independent endorsement by one female endorser, independent endorsement by four people, interdependent endorsement by one male endorser, interdependent endorsement by one female endorser, and dependent endorsement by four people. The subjects’ self-construal was first initiated, and after completing the task, they were asked two questions to test the effect of the manipulation: “What I just thought about made me think of myself” and “What I just thought about made me think of my friends/family” (Kühnen et al., 2001). The responses were scored on a 7-point Likert scale, with 1 indicating strong disagreement and 7 indicating strong agreement. The subjects then solved three simple arithmetic problems. They read the brand description of Benefactor car and viewed its promotional posters, followed by answering questions about their attitudes toward the brand. Finally, demographic information such as gender and age were collected. To conceal the purpose of our study, the researchers asked us to write down the number of brand endorsers they saw.

### 3.2 Results

#### 3.2.1 Reliability analysis

The reliability analysis results showed that Cronbach’s alpha coefficient values met the requirements for brand attitude (α = 0.96) and self-construal (α = 0.68).

#### 3.2.2 Manipulation test

The manipulation of self-construal was successful, as indicated by significant differences in the scores between independent and interdependent self-construal in both the independent self-construal initiation group (*M*_independent_ = 5.86, *M*_interdependent_ = 5.12, *F* (1, 255) = 7.015, *p* < 0.05, η^2^ = 0.027) and the interdependent self-construal initiation group (*M*_interdependent_ = 5.63, *M*_independent_ = 5.43, *F* (1, 255) = 7.362, *p* < 0.05, η^2^ = 0.028).

#### 3.2.3 Main effects

**Figure 2:**
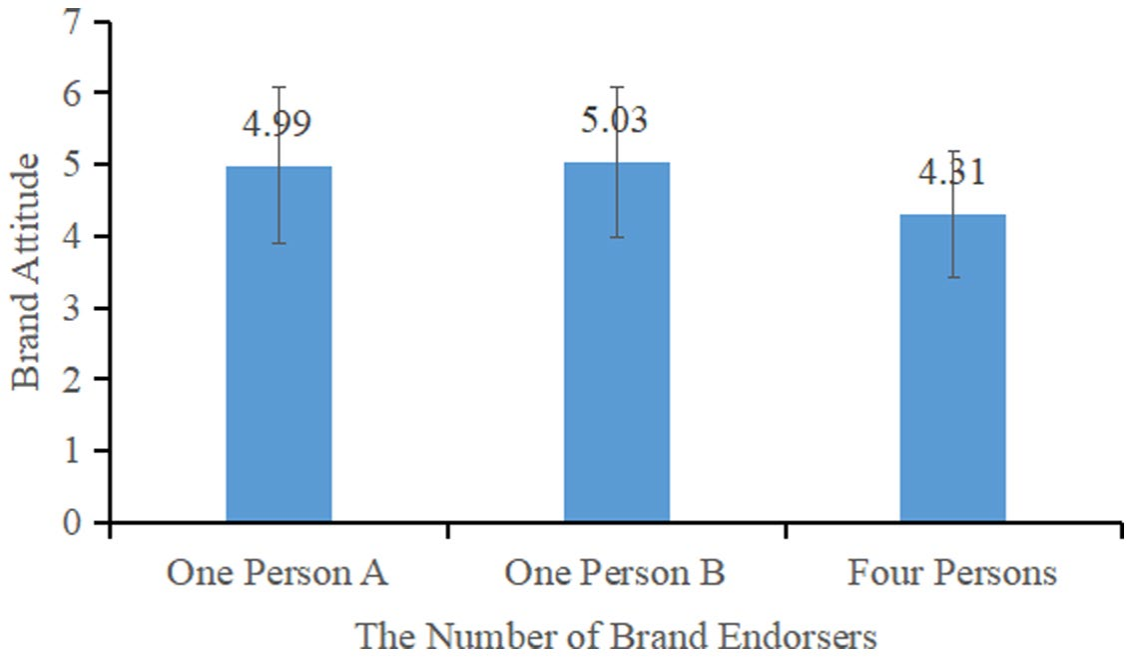
Effect of the number of brand endorsers on consumers’ brand attitudes.

The results of the main effect analysis showed a significant impact of the number of brand endorsers on brand attitude, *F* (2, 254) = 13.687, *p* = 0.000, η^2^ = 0.097. The subjects in the four-endorsers group (*M*_four_ = 4.31, SD = 0.88) had significantly lower brand attitudes compared to both the one person A endorser group (*M*_one person A_ = 4.99, SD = 1.08; *F* (1, 166) = 20.125, *p* = <0.001, η^2^ = 0.108) and the one person B endorsement group (*M*_one person B_ = 5.03, SD = 1.05; *F* (1, 169) = 20.782, *p* = <0.001, η^2^ = 0.123) (see Figure 2). There was no significant difference between the two one-person endorsement groups (*F* (1, 173) = 0.074, *p* = 0.786 > 0.05), validating Hypothesis 1 that the effect of single brand endorsement on consumers’ brand attitude was significantly higher than that of multiple brand endorsement.

#### 3.3.4 Moderated analysis

A univariate ANOVA was performed to examine the relationship between the number of brand endorsers, self-construal, and their interaction with consumers’ brand attitudes. The results showed a significant main effect of the number of brand endorsers on consumers’ brand attitudes (*F* (2, 254) = 14.023, *p* < 0.001). However, the main effect of self-construal (*F* (1, 255) = 1.856, *p* = 0.174) was not significant. The interaction between the number of brand endorsers and self-construal (*F* (2, 254) = 5.116, *p* < 0.05) was found to be significant. This indicates that the number of brand endorsers impacts consumers’ brand attitudes and that there is an interaction between the number of brand endorsers and self-construal.

**Figure 3:**
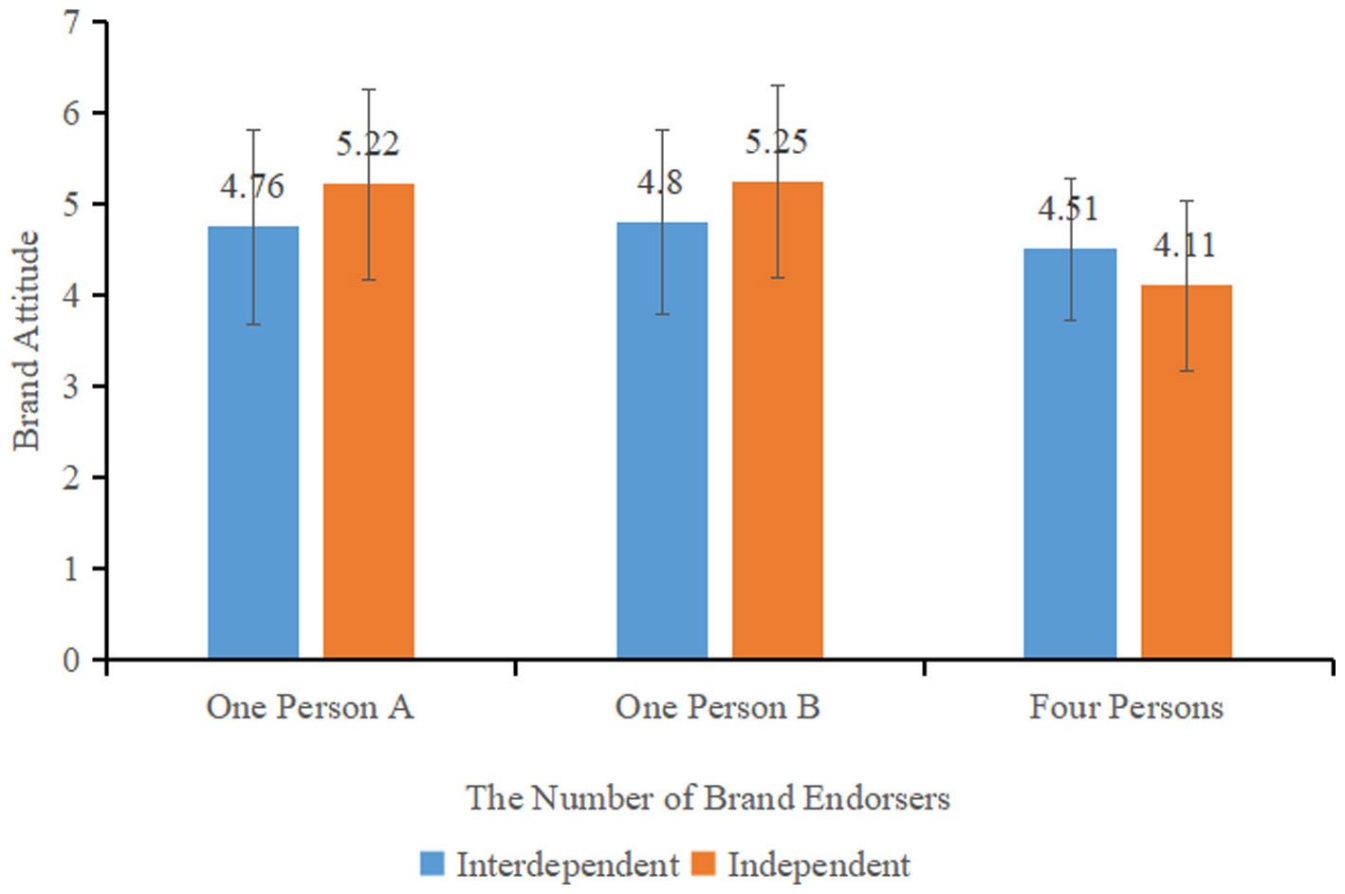
Effect of self-construal on consumers’ brand attitudes under different number of endorsers.

In the brand multiple (four-persons) endorsement group, consumers with interdependent self-construal (*M*_interdependent_ = 4.51, SD = 0.78) had higher brand attitudes compared to those with independent self-construal (*M*_independent_ = 4.11, SD = 0.94, *F* (1, 80) = 4.307, *p* < 0.05). In the brand one-person A group, consumers with independent self-construal (*M*_independent_ = 5.22, SD = 1.04) had higher brand attitudes compared to those with interdependent self-construal (*M*_interdependent_ = 4.76, SD = 1.07, *F* (1, 84) = 4.044, *p* < 0.05). In the brand one-person B group, consumers with independent self-construal (*M*_independent_ = 5.25, SD = 1.05) had higher brand attitudes compared to those with interdependent self-construal (*M*_interdependent_ = 4.80, SD = 1.01, *F* (1, 87) = 4.123, *p* < 0.05). These results, shown in Figure 3, indicate that under the moderation of self-construal, consumers with independent self-construal have more favorable brand attitudes toward single endorsement brands. In contrast, consumers with interdependent self-construal have more positive attitudes toward multiple endorsement brands, thus supporting Hypothesis 2.

### 3.3 Discussion

This experiment confirmed both Hypothesis 1 and 2. The results showed that the number of brand endorsers significantly impacts consumers’ brand attitudes, with consumers exhibiting more positive attitudes toward single-endorser brands. Additionally, the study found that self-construal moderates this relationship. The interaction between the number of brand endorsers and self-construal influences consumers’ brand attitudes. Consumers with an independent self-construal are more likely to prefer single-endorser brands, while consumers with an interdependent self-construal are more likely to prefer brands with multiple endorsers. To further test the robustness of Hypotheses 1 and 2, Study 2 used VR glasses as stimuli. However, the Western cultural regions from which the endorsers used in Study 1 came possessed different cultural backgrounds than the Eastern cultural region in which the subjects lived. It has been shown that cultural differences affect consumers’ information processing and that the type of race displayed in the endorser’s physical features and racial differences can affect the effectiveness of the endorsement (D’Alessandro and Chitty, 2011; Lord et al., 2019; Lee et al., 2021). Hence, unlike Study 1, we replaced the brand endorsers with Asian faces.

## 4 Study 2

Study 2 aimed to validate the findings from Study 1. The study design was a 3 × 2 factorial, with three brand endorsers (single female, single male, and four endorsers) and two levels of self-construal (independent and interdependent). The stimuli were VR glasses using the brand “VIVE,” a fictional brand designed to eliminate the influence of the real brand on consumers. The subjects were recruited from a community in Southern China and consisted of 203 individuals, including 130 women with an average age of 29.96 years (SD = 7.61).

### 4.1 Design and procedure

The study employed the Self-Construal Scale (SCS) developed by Singelis (1994), adapted to fit the cultural context in China. The SCS consisted of 17 items, including “I need to respect group decisions,” “I often feel that my relationships with others are more important than my achievements,” “I need to maintain the rapport of my group” and others, that were rated on a 7-point Likert scale, with 1 being “totally disagree” and 7 being “totally agree.” The scores for the two dimensions were obtained by taking the mean values of the corresponding scale items. The scores of the independent self-construal were subtracted from the interdependent self-construal and the centered difference means were used to assign participants to either the independent self-construal group (N = 98) or the interdependent self-construal group (*N* = 105).

The measurement of brand attitudes was based on Chattopadhyay and Basu’s (1990) brand attitudes scale, which included items such as “I like the brand,” “I agree with the brand,” “I think the brand is good,” and “I am satisfied with the brand.” These items were also scored on a 7-point Likert scale, with 1 being “totally disagree” and 7 being “totally agree.”

After the self-construal measurement, participants were asked to perform three simple arithmetic tasks, then randomly assigned to one of the three groups. Each group was presented with a description of the brand VIVE and a picture of its endorser, after which they rated their attitudes toward the brand. Finally, demographic information such as gender and age were collected from the participants.

### 4.2 Results

#### 4.2.1 Reliability analysis of self-construal

The self-construal analysis in this study was based on the study by Singelis (1994). The reliability analysis showed that the independent self-construal (α = 0.823) and the interdependent self-construal (α = 0.865) were measured at a high level of acceptability.

#### 4.2.2 Main effects

**Figure 4:**
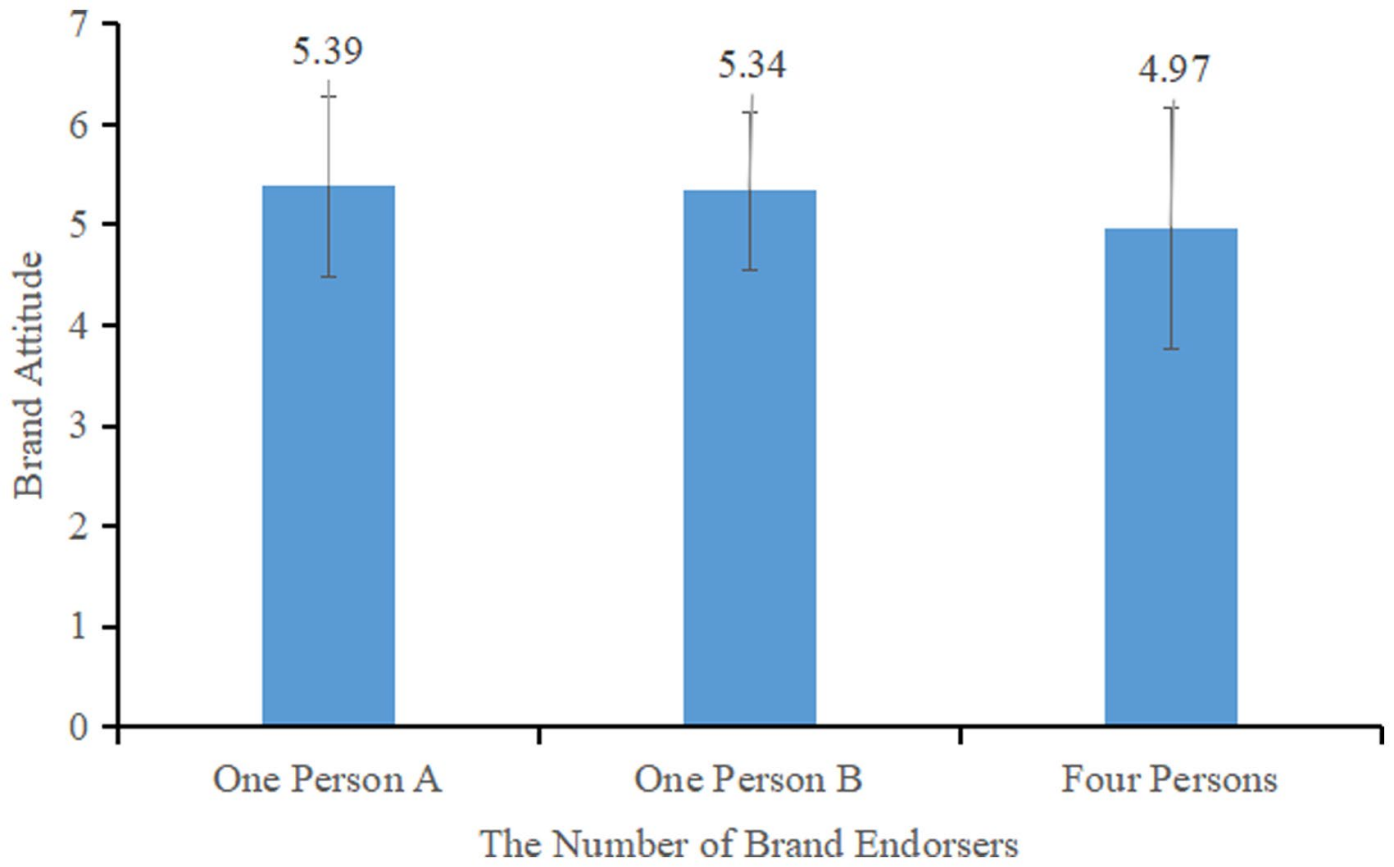
Effect of the number of brand endorsers on consumers’ brand attitudes.

The effect of the number of brand endorsers was significant, with *F* (2, 200) = 3.776, *p* < 0.05, η^2^ = 0.036. Participants in the brand four endorser group (*M*_four_ = 4.97, SD = 1.20) rated the brand significantly lower than both the brand one person A endorser group (*M*_one person A_ = 5.39, SD = 0.90; *F* (1,137) = 5.743, *p* < 0.05, η^2^ = 0.040) and the brand one person B endorsement group (*M*_one Person B_ = 5.34, SD = 0.78; *F* (1,125) = 4.282, *p* < 0.05, η^2^ = 0.033). There was no significant difference between the two single endorsement groups (*F* (1,138) = 0.146, *p* = 0.703, η^2^ = 0.001) as shown in Figure 4. These results indicate that the effect of single-brand endorsement on consumers’ brand attitude is significantly higher than that of multi-brand endorsement, supporting Hypothesis 1.

#### 4.2.3 Moderated analysis

The moderated analysis of the study explored the relationship between the number of brand endorsers, self-construal, and their impact on consumers’ brand attitudes. Results showed that the effect of the number of brand endorsers on brand attitudes was significant (*F* (2, 197) = 6.707, *p* < 0.005), while the effect of self-construal (*F* (1, 197) = 0.036, *p* = 0.849) was not significant. However, the interaction between the number of brand endorsers and self-construal was significant (*F* (2, 197) = 52.188, *p* < 0.001). This suggests that the number of brand endorsers and self-construal interact to impact consumers’ brand attitudes.

**Figure 5:**
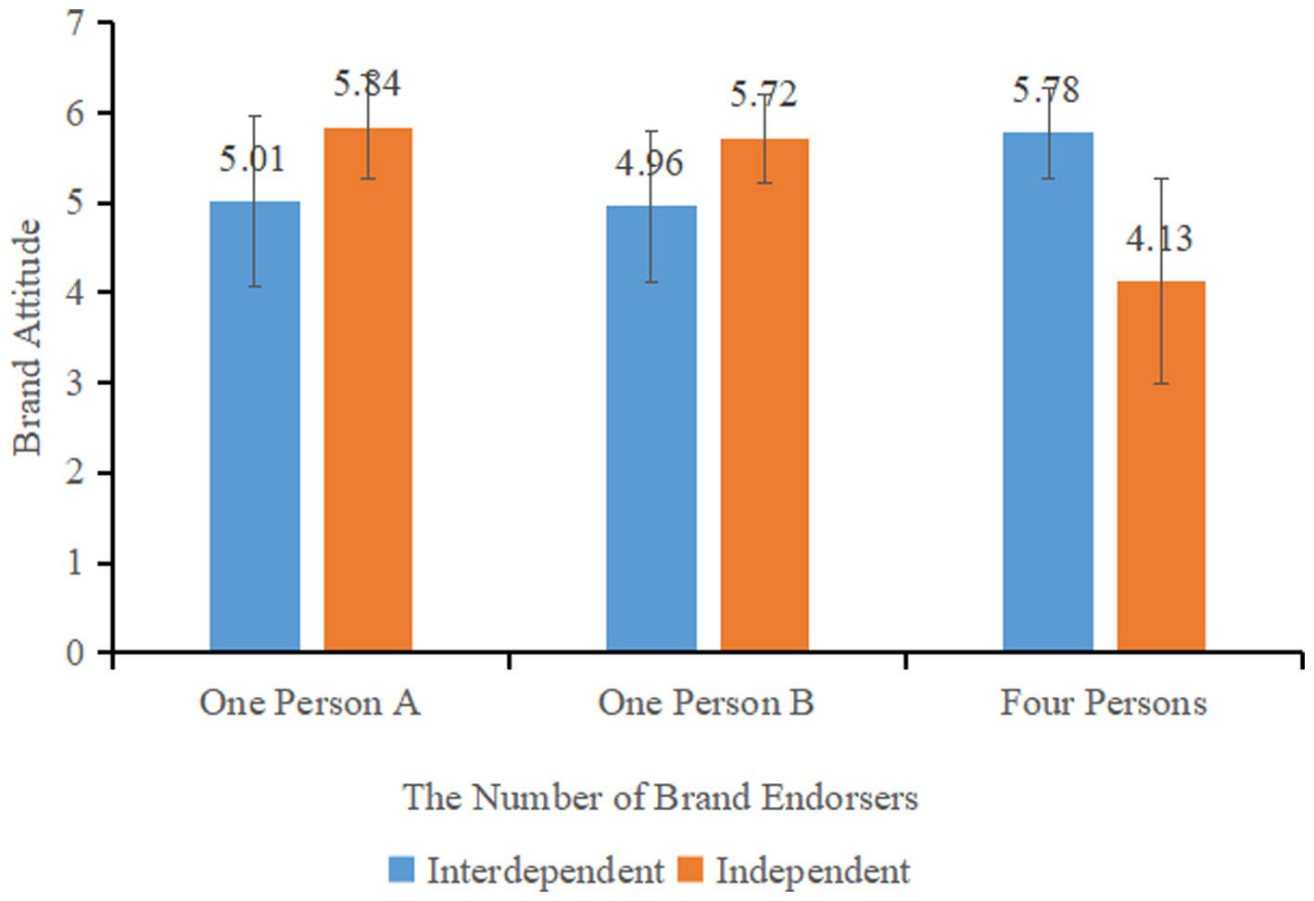
Effect of self-construal on consumers’ brand attitudes under different number of endorsers.

For consumers in the multiple (four-person) endorsement group, those with interdependent self-construal (*M*_interdependent_ = 5.78, SD = 0.51) had higher brand attitudes compared to those with independent self-construal (*M*_independent_ = 4.13, SD = 1.13, *F* (1, 61) = 56.597, *p* < 0.001, ƞ^2^ = 0.481). In the single (one-person A) endorsement group, consumers with independent self-construal (*M*_independent_ = 5.84, SD = 0.58) had higher brand attitudes compared to those with interdependent self-construal (*M*_interdependent_ = 5.01, SD = 0.95, *F* (1, 74) = 20.456, *p* < 0.001, ƞ^2^ = 0.217). Similarly, in the single (one person B) endorsement group, consumers with independent self-construal (*M*_independent_ = 5.72, SD = 0.49) had higher brand attitudes compared to those with interdependent self-construal (*M*_interdependent_ = 4.96, SD = 0.85, *F* (1, 62) = 19.323, *p* < 0.001, ƞ^2^ = 0.238) (as shown in Figure 5). These results indicate that, as moderated by self-construal, consumers with independent self-construal have higher brand attitudes toward single endorsement brands, while those with interdependent self-construal have higher brand attitudes toward multiple endorsement brands, and Hypothesis 2 is confirmed.

### 4.3 Discussion

Study 2 tested Hypothesis 1 and Hypothesis 2 using a different product brand (VR glasses) from Study 1. The results confirmed that the number of brand endorsers impacts consumers’ brand attitudes. Consumers with independent self-construal tend to have higher brand attitudes toward single endorser brands. Consumers with interdependent self-construal tend to have higher brand attitudes toward multiple endorser brands. Unlike the products with both hedonic and utilitarian attributes (cars and VR glasses) used in Studies 1 and 2 (Chitturi et al., 2008; Herz and Rauschnabel, 2019), Study 3 used a hedonic product (cosmetics) (Bourguet et al., 2016). The robustness of Hypotheses 1 and 2 is further tested and the generalization of this effect across brand endorsement marketing campaigns is expanded. With the support of digital technology, virtual image endorsers are popular in advertising because of their unique advantages (Lv et al., 2023). In Study 3, five existing virtual influencers were selected as endorsers to verify whether the effects generated on real-person endorsers can extend to virtual endorsers. The interference of consumers’ liking and familiarity with the brand endorser was not excluded in the previous studies. Therefore, Study 3 excluded the interference of endorser liking and familiarity by adding a pre-test.

## 5 Study 3

Study 3 aimed to confirm the results from Study 1 and 2 and examine the mediating effect of self-consistency (H3). A real cosmetics brand, Shiseido, was used as the stimulus. Moreover, we used Shiseido’s own adopted virtual digital person endorsers as the stimulus materials. A total of 247 participants from various backgrounds were recruited online, with 144 of them being women with an average age of 28.68 years (SD = 7.12). The procedure and measurement scale were like Study 1, using a 3 (number of brand endorsers: single endorser vs. two vs. five) × 2 self-construal (independent vs. interdependent) between-group design. A multi-person group (two vs. five) was added as a control in selecting the number of brand endorsers to eliminate the effect of the difference in the number of multi-person endorsers.

### 5.1 Pre-test

A total of 250 participants from Southern China participated in a pre-test. They viewed a picture of the endorser and then rated their liking and familiarity using two questions: “How much do you like the person in the picture” (rated on a scale from 1 = “very much dislike” to 7 = “very much”) and “How familiar are you with the person in the picture” (rated on a scale from 1 = “very unfamiliar” to 7 = “very familiar”). The results of the manipulation test showed that:

There was no significant difference in the subjects’ liking of the 5 endorsers (*F* (4, 1,245) = 0.890, *p* = 0.578 > 0.05; M_1_ = 4.98, SD = 1.30; M_2_ = 5.00, SD = 1.33; M_3_ = 4.95, SD = 1.30; M_4_ = 5.03, SD = 1.36; M_5_ = 4.91, SD = 1.42). Furthermore, there was no significant difference in the familiarity of the subjects with the 5 endorsers, and all 5 endorsers had low familiarity (*F* (4, 1,245) = 0.705, *p* = 0.588 > 0.05; M_1_ = 3.89, SD = 1.65; M_2_ = 4.08, SD = 1.82; M_3_ = 4.09, SD = 1.69; M_4_ = 4.08, SD = 1.80; M_5_ = 3.95, SD = 1.77). In conclusion, there is a slight variation in the popularity and familiarity of the 5 brand endorsers.

### 5.2 Design and procedure

The Self-construal manipulation experiment involved 247 participants recruited from Credamo. The study aimed to initiate the subjects’ self-construal using a guidance grammar, drawing on Trafimow et al.’s (1991) study. Participants were given a 3-min time frame to think and write down their answers based on the guiding phrase assigned to them. The independent self-construal group was instructed, “Please think about what is expected of you.” In contrast, the interdependent self-construal group was instructed, “Please think about what is expected of you by your family or friends.”

The participants were then evaluated on two measures, self-consistency, and brand attitudes. The self-consistency evaluation was based on a 7-point scale (Escalas and Bettman, 2003, 2005) to measure association with a particular brand, with questions like “The brand Larissa reflects who I am,” “I identify with the brand Larissa,” and “I feel an emotional connection to Larissa.” The brand attitudes evaluation was based on Chattopadhyay and Basu’s (1990) brand attitudes scale, with four items: “I like the brand,” “I approve of the brand,” “I think the brand is good,” and “I am satisfied with the brand.” Both evaluations were scored using a 7-point Likert scale, where 1 means “totally disagree”, and 7 means “totally agree.”

The procedure started by assessing the effect of the manipulation by asking the participants two questions: “What I just thought made me think of myself” and “What I just thought made me think of my friends/family.” The subjects were then randomly assigned to one of three groups and shown a promotional video about the car brand Lion advertisement. After viewing the video, participants rated the brand’s attitude and self-consistency. At the end of the study, the subjects’ basic information, such as gender and age, was recorded.

## 6 Results

### 6.1 Self-construal manipulation

The results of the self-construal manipulation showed a significant difference between the independent and interdependent self-construal scores for both initiation groups. The scores of independent self-construal were higher in the independent self-construal initiation group (*M* = 6.22) compared to the interdependent self-construal group (*M* = 5.00), *F* (1, 245) = 32.338, *p* < 0.001, η^2^ = 0.117. On the other hand, the scores of interdependent self-construal were higher in the interdependent self-construal initiation group (*M* = 6.15) compared to the independent self-construal group (*M* = 5.33), *F* (1, 245) = 34.690, *p* < 0.001, η^2^ = 0.124, indicating that the self-construal manipulation was effective.

### 6.2 Main effects

**Figure 6:**
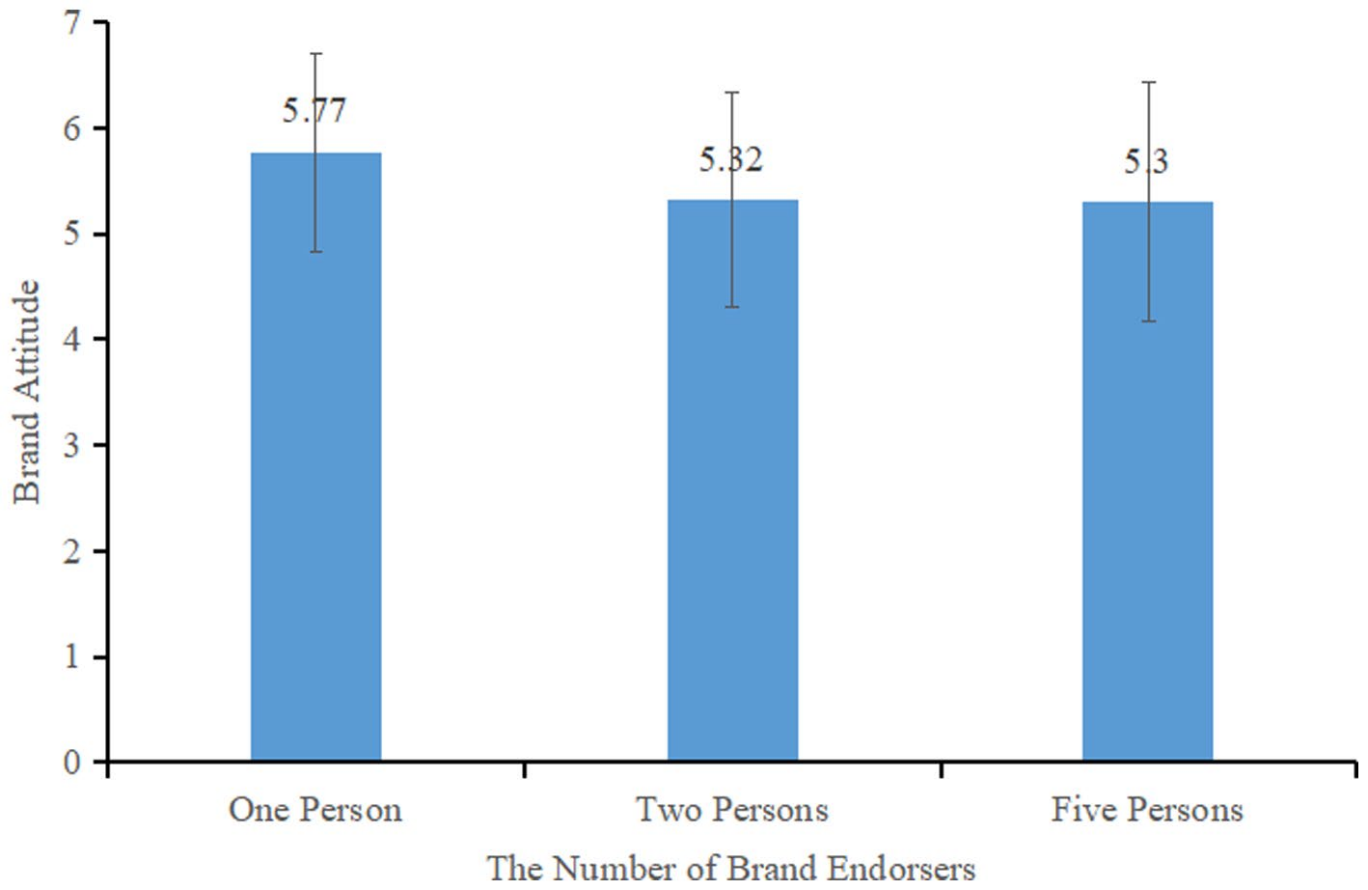
Effect of the number of brand endorsers on consumers’ brand attitudes.

The study found a significant main effect of the number of brand endorsers on consumers’ brand attitudes, *F* (2, 244) = 5.371, *p* < 0.05, η^2^ = 0.042. Brand attitudes were higher in the group with one brand endorser (*M* = 5.77, SD = 0.94) compared to the group with two endorsers (*M* = 5.32, SD = 1.02) and the group with five endorsers (*M* = 5.30, SD = 1.13) (see Figure 6), with both showing significant differences, *F* (1, 165) = 8.683, *p* < 0.05 and *F* (1, 157) = 8.308, *p* < 0.05, respectively. However, there was no significant difference between the groups with two and five endorsers, *F* (1, 166) = 0.023, *p* = 0.880. These results support Hypothesis 1, which states that the effect of single brand endorsement on consumers’ brand attitude is higher than that of multiple brand endorsement.

### 6.3 Moderation analysis

**Figure 7:**
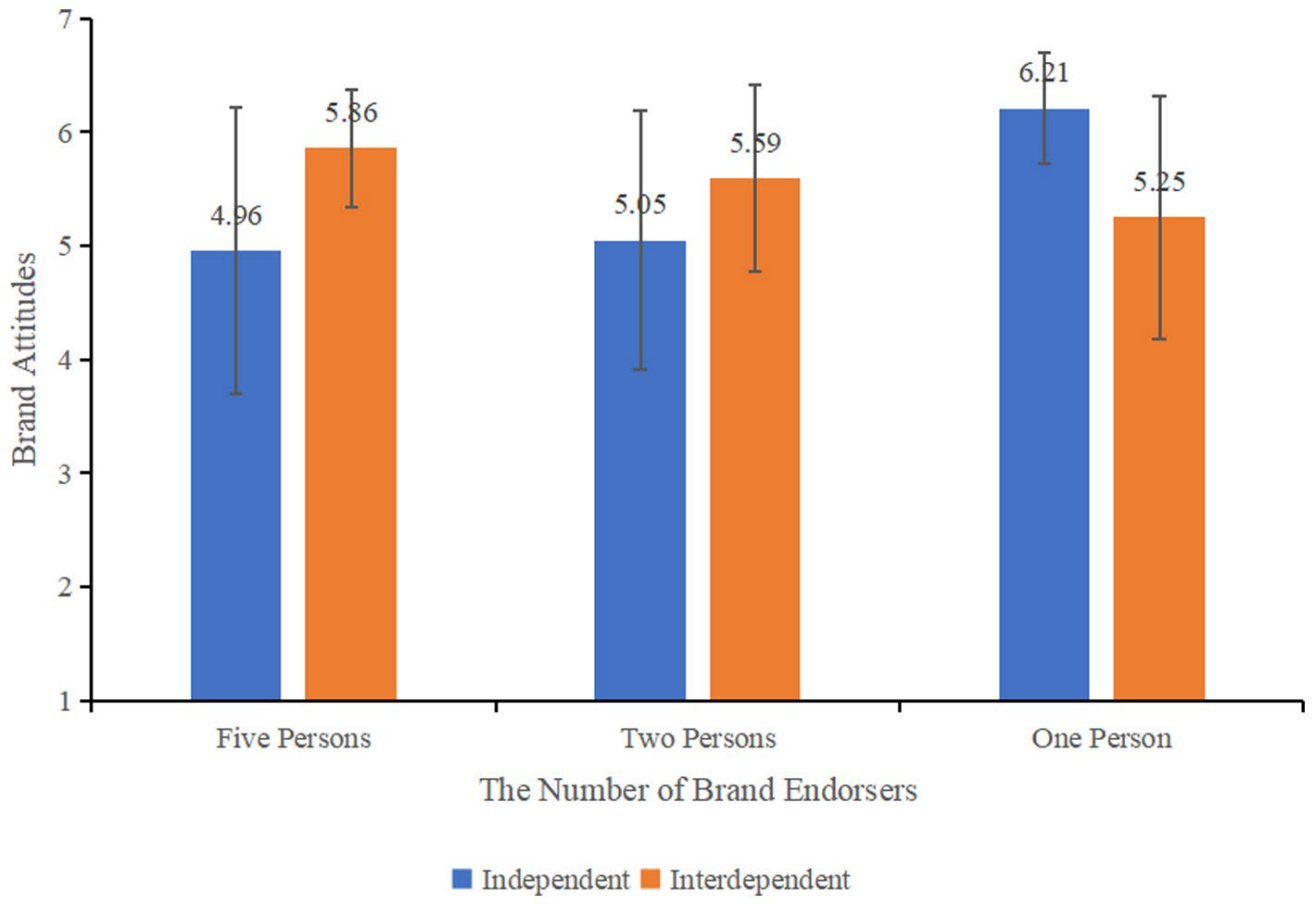
Effect of self- construal on consumers’ brand attitudes under different number of endorsers.

A univariate ANOVA was performed to examine the relationship between the number of brand endorsers, self-construal, and their impact on consumers’ brand attitudes. The results indicated that the number of brand endorsers had a significant effect on consumers’ brand attitudes (*F* (2, 241) = 5.082, *p* < 0.05). In contrast, the main effect of self-construal (*F* (1, 241) = 2.470, *p* = 0.117) was not significant. However, the interaction between the number of brand endorsers and self-construal had a significant impact on consumers’ brand attitudes (*F* (2, 241) = 18.319, *p* < 0.001). In the group of five brand endorsers, consumers with an interdependent self-construal (*M*_interdependent_ = 5.86, SD = 0.52) had higher brand attitudes than those with an independent self-construal (*M*_independent_ = 4.96, SD = 1.26, *F* (1, 78) = 14.083, *p* < 0.001). A similar trend was observed in the two-endorser group and the one-endorser group, where interdependent self-construal had a positive effect on brand attitudes, while independent self-construal had a negative effect (as shown in Figure 7). This suggests that self-construal moderates the relationship between the number of brand endorsers and consumers’ brand attitudes, thereby supporting Hypothesis 2.

### 6.4 Mediated moderation analysis

The mediating role of self-consistency was explored using the mediated regulation model (Model 7) proposed by Hayes (2018), with a sample size of 5,000. The number of brand endorsers was set as the independent variable, self-construal as the moderating variable, and self-consistency as the mediating variable. Results showed that self-consistency played a significant mediating role in the relationship between the number of brand endorsers, self-construal, and consumers’ brand attitudes (undirected path effect = −0.56, SE = 0.13, 95% CI: [−0.8237, −0.3191], excluding 0). Specifically, self-consistency was a significant mediator for both the independent self-construal group (effect = −0.37, SE = 0.10, 95% CI: [−0.5634, −0.1790], excluding 0) and the interdependent self-construal group (effect = 0.19, SE = 0.08, 95% CI: [0.0465, 0.3536], excluding 0). This suggests that self-consistency mediates the relationship between the number of brand endorsements and self-construal on consumers’ brand attitudes, with independent self-construal being consistent with a single endorsement and interdependent self-construal being consistent with multiple endorsements.

## 7 Discussion

Study 3 showed that the number of brand endorsers influenced consumers’ brand attitudes and that this effect was moderated by self-construal. It further verified that this effect was also present when the endorser was a virtual digital person. Consumers with independent self-construal had more favorable attitudes toward brands endorsed by one person. In contrast, consumers with interdependent self-construal had more favorable attitudes toward brands endorsed by multiple people. The results also indicated that self-consistency significantly mediated the interaction between the number of brand endorsers and self-construal on consumers’ brand attitudes.

## 8 Conclusion

The current study examines the impact of the self-construal and its interaction with the number of brand endorsers in consumer brand attitudes. Based on previous research on the number of brand endorsers (Tripp et al., 1994; Rice et al., 2012; Wang et al., 2015; Handriana, 2017) and the types of consumer self-construal (White et al., 2012; Sugitani, 2018; Schouten et al., 2020), the paper uses self-construal theory to explore the relationship between the number of brand endorsers and consumer brand attitudes. We hypothesize that in general, a single endorser’s endorsement strategy leads to more positive brand attitudes among consumers. However, according to self-construal theory, when consumers are in collectivist culturally inclined areas, the interdependent self-construal they possess will have more positive brand attitudes toward multi-person endorsements; when consumers are in individualist culturally inclined areas, the independent self-construal they possess will have more positive brand attitudes toward single-person endorsements. And self-consistency mediates this effect. Three studies supported the above hypothesis. Three studies confirmed the robustness of the hypotheses by replacing advocates with different characteristics. On the basis that the subjects were all from Asian cultural areas, Study 1 used endorsers from different cultural backgrounds with Western cultural regions; Study 2 used endorsers from the same cultural backgrounds with Asian facial features; and Study 3 used virtual digital people designed based on Asian facial features as endorsers to extend the connotation of endorsers further.

### 8.1 Theoretical contributions

Firstly, the present paper adds to the existing body of research on the relationship between the number of brand endorsers and consumer brand attitudes. While previous studies have primarily focused on single-endorser brands, Rice et al. (2012) have noted that multiple brand endorsements by a single individual have become common. This study expands upon these findings by exploring the impact of the number of brand endorsers on consumer brand attitudes in single and multiple endorsement scenarios. The results indicate that consumers favor and exhibit positive attitudes toward a single brand endorsement.

Secondly, this paper introduces the concept of self-construal theory and posits boundary conditions under which the number of brand endorsers can influence consumers’ brand attitudes. The paper thus contributes to our understanding of the factors that shape brand attitudes. While previous research has focused on external factors such as brand story type (Avery et al., 2010), brand logo type (Labrecque and Milne, 2012), and brand crisis type (Sinha and Lu, 2016), the influence of cultural factors has been understudied. This paper bridges this gap by showing that the number of brand endorsers and consumers’ self-construal (independent vs. interdependent) interact to impact brand attitudes. Results suggest that consumers with independent self-construal prefer single-endorser brands, while those with interdependent self-construal are more likely to choose brands endorsed by multiple individuals.

Finally, this paper posits that self-consistency is a mediator between the number of brand endorsers, self-construal, and consumers’ brand attitudes, adding to our knowledge of self-consistency. Previous studies have explored the relationship between brands and self-consistency in explaining the impact of brand and celebrity type on consumer attitudes (Zhu et al., 2019). Steele and Liu (1983) noted that individuals’ self-protection drives brand word-of-mouth, and many studies have established that the match between brand image and consumers’ self-image significantly affects brand attitudes. This paper enriches the understanding of brand-consumer congruence by providing a self-construal perspective and offering new research insights.

### 8.2 Practical contributions

This paper provides valuable insights for companies to develop a strategy for the number of brand endorsers. First, brand endorsers represent the brand’s image and identity (Elberse and Verleun, 2012). The study shows that for a market that tends to be an individualistic culture, consumers are mainly independent, when companies use a single endorser is more conducive to improving brand attitudes; for a market that tends to be a collectivistic culture, consumers are mainly interdependent, when companies use multiple endorsers is more conducive to enhancing brand attitudes，so it is critical to develop strategies for different numbers of endorsers for markets with different cultural backgrounds. Second, when the image of the brand endorser has a high degree of consumer self-consistency, it is conducive to bringing consumers closer to the brand’s psychological distance (Zogaj et al., 2021). This study proves that self-consistency mediates the effect of the number of endorsers on brand attitudes. Consequently, companies can adjust the number of endorsers for different cultural markets to increase consumers’ self-consistency, improve their brand attitudes and bridging the brand relationship. Finally, previous research on self-construal in social media environments shows that interdependent consumers express more positive attitudes toward social media posts (Hofmann et al., 2021). Therefore, in areas where collectivist culture is prevalent, companies should use multiple endorsers for their social media marketing campaigns. In addition, this study demonstrates that the number of endorsers strategy is effective for both human endorsers and virtual endorsers (e.g., virtual influencers, virtual digital humans), so cultural industries that use virtual endorsers as their primary endorsers may also consider adapting their endorser promotion strategies.

### 8.3 Research limitations and future work

This study only explores the impact of the number of brand endorsers on consumers’ brand attitudes through the lens of self-construal, ignoring other moderating factors. Other studies have found that the type of brand endorser (celebrities vs. social media influencers) (Zhu et al., 2022) and the type of brand endorser (real vs. virtual endorsers) (Zhu et al., 2019) also influences consumers’ brand attitudes. In addition, cultural variables such as power perception (Liang et al., 2023), risk preference (Liang et al., 2022), and social exclusion (Liang et al., 2021) also affect consumers’ brand attitudes. Hence, future research should consider these factors. Additionally, the interaction between self-construal and the number of brand endorsers in this paper is mediated by self-consistency, but the degree of self-consistency (high vs. low) and the effect of self-concept (real self vs. ideal self) (Zhu et al., 2019) have not been considered. Future studies can make a more nuanced division of self-consistency to examine the influence of self-consistency and self-construal (independent vs. interdependent) or the effect of self-consistency (real self vs. ideal self).

## Data availability statement

The datasets presented in this article are not readily available because the dataset contains the subjects’ addresses and IP addresses. Requests to access anonymized datasets should be directed to the corresponding author.

## Ethics statement

The studies involving humans were approved by the Ethics Committee of Guangxi University. The studies were conducted in accordance with the local legislation and institutional requirements. The participants provided their written informed consent to participate in this study.

## Author contributions

SL: Conceptualization, Funding acquisition, Methodology, Writing – review & editing. KC: Data curation, Investigation, Writing – original draft. YZ: Data curation, Formal analysis, Investigation, Methodology, Software, Supervision, Visualization, Writing – original draft. XY: Software, Supervision, Visualization, Writing – review & editing. SP: Funding acquisition, Supervision, Writing – review & editing. LT: Supervision, Validation, Writing – review & editing.
