# Correction: One independent or many independent? The relationship among self-construal, number of brand endorsers and brand attitudes

**DOI:** 10.3389/fpsyg.2025.1654914

**Published:** 2025-07-18

**Authors:** Shichang Liang, Kunhan Cai, Yiwei Zhang, Xueying Yuan, Siyu Pan, Lili Teng

**Affiliations:** ^1^School of Business, Guangxi University, Nanning, Guangxi, China; ^2^China-Asean Institute of Financial Cooperation, Guangxi University, Nanning, Guangxi, China; ^3^Business School, Central South University, Changsha, Hunan, China

**Keywords:** brand endorser, number of endorsers, self-construal, brand attitude, self-consistency

In the published article, there was an error in Figure 7 as published. Figure 5 and Figure 7 were erroneously assigned the same image, and the correct image for Figure 7 was not uploaded. The corrected Figure 7 caption should be: Effect of self-construal on consumers' brand attitudes under different number of endorsers.

The correct Figure 7 appears below.

In the published article, there was an error. There were missing symbols for statistical result parameters in the main body text.

A correction has been made to **4.2 Results**, *4.2.3 Moderated analysis*, Paragraph 2. The sentences previously stated:

“For consumers in the multiple (four-person) endorsement group, those with interdependent self-construal (M interdependent = 5.78, SD = 0.51) had higher brand attitudes compared to those with independent self-construal (M _independent_= 4.13, SD = 1.13, F (1,61) = 56.597, *p* < 0.001, = 0.481).”

“In the single (one-person A) endorsement group, consumers with independent self-construal (M _independent_= 5.84, SD = 0.58) had higher brand attitudes compared to those with interdependent self-construal (M _interdependent_= 5.01, SD = 0.95, F (1,74) = 20.456, *p* < 0.001, = 0.217).”

“Similarly, in the single (one person B) endorsement group, consumers with independent self-construal (M _independent_=5.72, SD = 0.49) had higher brand attitudes compared to those with interdependent self-construal (M _interdependent_= 4.96, SD = 0.85, F (1,62) = 19.323, *p* < 0.001, = 0.238) (as shown in Figure 5)”

The corrected sentences appear below:

“For consumers in the multiple (four-person) endorsement group, those with interdependent self-construal (M interdependent = 5.78, SD = 0.51) had higher brand attitudes compared to those with independent self-construal (M _independent_ = 4.13, SD = 1.13, F_(1, 61)_ = 56.597, *p* < 0.001, η^2^ = 0.481).”

“In the single (one-person A) endorsement group, consumers with independent self-construal (M _independent_ = 5.84, SD = 0.58) had higher brand attitudes compared to those with interdependent self-construal (M _interdependent_ = 5.01, SD = 0.95, F_(1, 74)_ = 20.456, *p* < 0.001, η^2^ = 0.217).”

“Similarly, in the single (one person B) endorsement group, consumers with independent self-construal (M _independent_ = 5.72, SD = 0.49) had higher brand attitudes compared to those with interdependent self-construal (M _interdependent_ = 4.96, SD = 0.85, F_(1, 62)_ = 19.323, *p* < 0.001, η^2^ = 0.238) (as shown in Figure 5)”.

**Figure 7:**
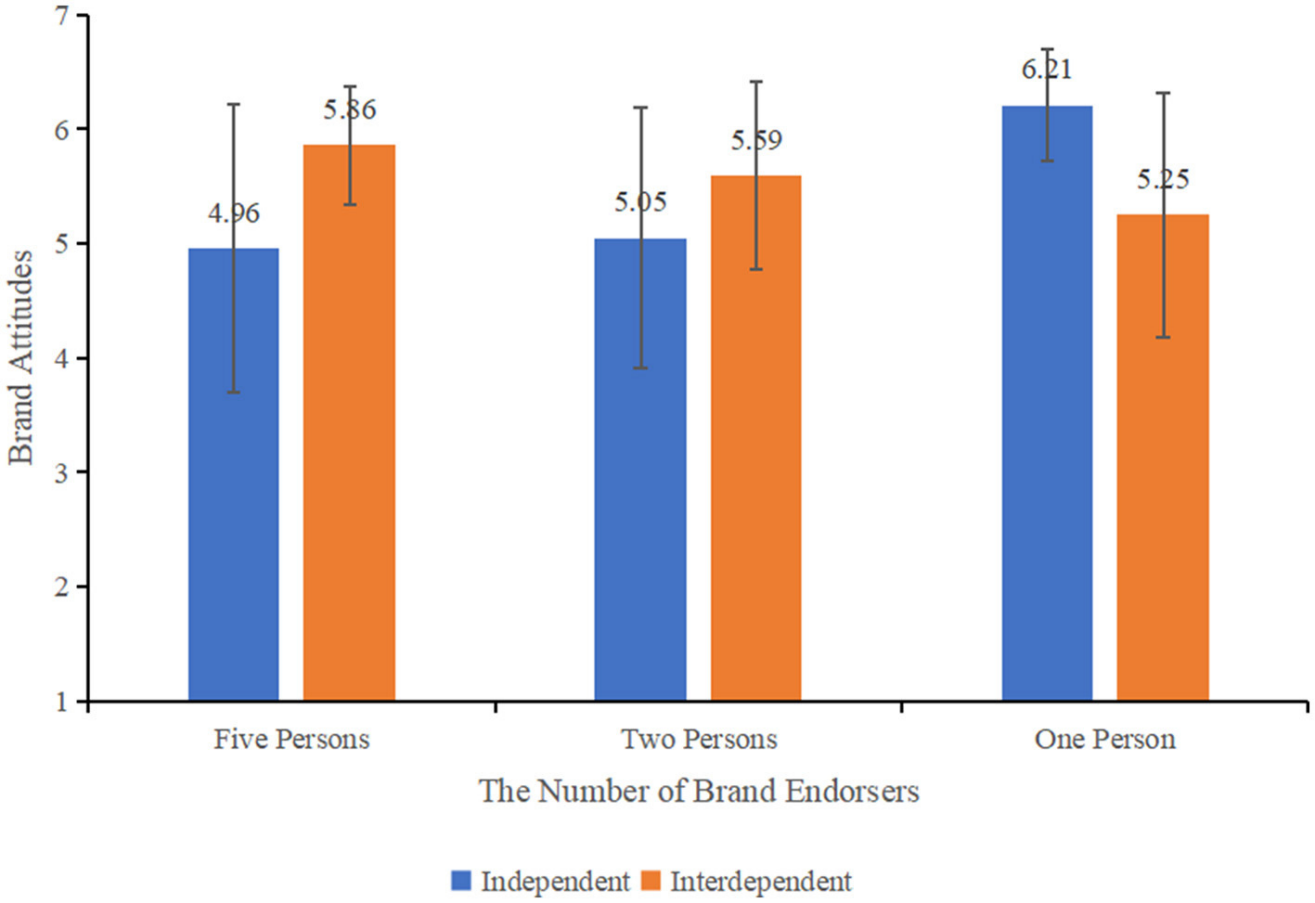
Effect of self-construal on consumers' brand attitudes under different number of endorsers.

In the published article, there was an error. The phrase “In addition, cultural variables such as power perception, risk preference, and social exclusion also affect consumers' brand attitudes.” appears twice consecutively.

A correction has been made to **8.3 Research limitations and future work**. This sentence previously stated:

“In addition, cultural variables such as power perception, risk preference, and social exclusion also affect consumers' brand attitudes. In addition, cultural variables such as power perception (Liang et al., 2023), risk preference (Liang et al., 2022), and social exclusion (Liang et al., 2021) also affect consumers' brand attitudes.”

The corrected sentence appears below:

“In addition, cultural variables such as power perception (Liang et al., 2023), risk preference (Liang et al., 2022), and social exclusion (Liang et al., 2021) also affect consumers' brand attitudes.”

The original article has been updated.

